# Current clinical practice of deep brain stimulation (DBS) for treatment-refractory obsessive compulsive disorder (OCD): a multi-center survey of five European DBS for OCD programs

**DOI:** 10.1192/j.eurpsy.2026.10250

**Published:** 2026-07-31

**Authors:** M. Naesström, R. Mocking, D. Denys, R. Schuurman, J. C. Baldermann, D. Meyer-Doll, M. Polosan, S. Chabardes, C. Bervoets, H. Heylen, B. Nuttin, P. Blomstedt, V. Coenen

**Affiliations:** 1 Department of Clinical Sciences, Psychiatry, Umeå University, Umeå, Sweden; 2 Department of Psychiatry; 3 Department of Neurosurgery, Amsterdam UMC, Amsterdam, Netherlands; 4 Department of Psychiatry and Psychotherapy, University of Freiburg, Freiburg, Germany; 5 Department of Psychiatry; 6 Department of Neurosurgery, Centre Hospitalier universitaire Grenoble Alpes, Grenoble, France; 7 Psychiatry; 8 Neurosurgery, University Hospitals Leuven, Leuven, Belgium; 9 Department of Clinical Sciences, Neurosurgery, Umeå University, Umeå, Sweden; 10 Department of Stereotactic and Functional Neurosurgery, University of Freiburg, Freiburg, Germany

## Abstract

**Introduction:**

Deep Brain Stimulation (DBS) is a treatment option for severe, treatment-refractory (TR) obsessive-compulsive disorder (OCD). Yet, real-world practices may vary across international centers potentially resulting in a variability of treatment outcomes.

**Objectives:**

To describe (i) eligibility criteria and definitions of TR OCD to qualify for DBS (ii) DBS targets (iii) governance/referral processes, and (iv) pre- and post-operative management—including programming, adverse-event handling, and rehabilitation—across five European programs of DBS for TR-OCD.

**Methods:**

As an initiative of the Psychiatric Surgery Task Force of the World Society of Stereotactic and Functional Neurosurgery a questionnaire was sent to selected international DBS centers with a dedicated psychiatric team asking detailed questions regarding patient selection, experience and management of patients treated with DBS for TR OCD. Here we report the results from the participating European centers.

**Results:**

Five programs(Amsterdam, Freiburg, Grenoble, Leuven, Umeå) reported a total experience of 241 patients with severe TR OCD treated with DBS. Targets included nucleus accumbens, ventral/anterior limb of the internal capsule, superolateral medial forebrain bundle/ventral tegmental area, anteromedial subthalamic nucleus and the bed nucleus of the stria terminalis.

Common inclusion criteria included age over 18 years, primary OCD with marked severity and chronicity ranging from severe OCD on the Yale-Brown Obsessive Compulsive scale, with a minimum disease duration of ≥5 years and capacity to consent to the treatment.

Definitions of TR OCD were similar across the centers: unsuccessful trials of ≥2 selective serotonin reuptake inhibitors plus clomipramine, antipsychotic augmentation, and cognitive behavioural therapy with exposure/response prevention.

Common exclusion criteria across the centers included psychosis, major surgical/medical contraindications, significant central nervous system disease/trauma, active substance misuse, severe personality disorder, and acute suicidality. Accepted psychiatric comorbidities varied slightly between centers where some allowed for comorbidity e.g with anorexia nervosa.

All centers mentioned psychotherapy as part of the post-surgical rehabilitation

**Conclusions:**

Overall the definition of TR OCD and inclusion criteria for DBS were harmonized across multiple centers in Europe. DBS may be considered for selected patients with severe, TR OCD and should be provided by a dedicated, multidisciplinary team with psychiatric expertise.

**Disclosure of Interest:**

None Declared

